# DeepSeek in Healthcare: Revealing Opportunities and Steering Challenges of a New Open-Source Artificial Intelligence Frontier

**DOI:** 10.7759/cureus.79221

**Published:** 2025-02-18

**Authors:** Abdulrahman Temsah, Khalid Alhasan, Ibraheem Altamimi, Amr Jamal, Ayman Al-Eyadhy, Khalid H Malki, Mohamad-Hani Temsah

**Affiliations:** 1 Software Engineering, Alfaisal University, Riyadh, SAU; 2 Pediatric Department, College of Medicine, King Saud University, Riyadh, SAU; 3 Kidney and Pancreas Health Center, Organ Transplant Center of Excellence, King Faisal Specialist Hospital and Research Center, Riyadh, SAU; 4 Evidence-Based Research Chair, Family and Community Medicine, College of Medicine, King Saud University, Riyadh, SAU; 5 Pediatric Intensive Care Unit, Pediatric Department, King Saud University Medical City, Riyadh, SAU; 6 Research Chair of Voice, Swallowing, and Communication Disorders, Department of Otolaryngology-Head and Neck Surgery, College of Medicine, King Saud University, Riyadh, SAU

**Keywords:** ai hallucinations and bias in medicine, deepseek deepthink r1 open-source ai, ethical considerations in ai-driven healthcare, generative artificial intelligence (gai) in healthcare, healthcare data privacy and ai compliance, hipaa and gdpr ai compliance, large language models (llms) in medicine, medical education and ai integration, offline ai deployment for healthcare, open-source ai for medical research

## Abstract

Generative Artificial Intelligence (GAI) has driven several advancements in healthcare, with large language models (LLMs) such as OpenAI’s ChatGPT, Google’s Gemini, and Microsoft’s Copilot demonstrating potential in clinical decision support, medical education, and research acceleration. However, their closed-source architecture, high computational costs, and limited adaptability to specialized medical contexts remained key barriers to universal adoption. Now, with the rise of DeepSeek’s DeepThink (R1), an open-source LLM, gaining prominence since mid-January 2025, new opportunities and challenges emerge for healthcare integration and AI-driven research. Unlike proprietary models, DeepSeek fosters continuous learning by leveraging publicly available open-source datasets, possibly enhancing adaptability to the ever-evolving medical knowledge and scientific reasoning. Its transparent, community-driven approach may enable greater customization, regional specialization, and collaboration among data researchers and clinicians.

Additionally, DeepSeek supports offline deployment, addressing some data privacy concerns. Despite these promising advantages, DeepSeek presents ethical and regulatory challenges. Users’ data privacy worries have emerged, with concerns about user data retention policies and potential developer access to user-generated content without opt-out options. Additionally, when used in healthcare applications, its compliance with China’s data-sharing regulations highlights the urgent need for clear international data privacy and governance. Furthermore, like other LLMs, DeepSeek may face limitations related to inherent biases, hallucinations, and output reliability, which warrants rigorous validation and human oversight before clinical application. This editorial explores DeepSeek’s potential role in clinical workflows, medical education, and research while also highlighting its challenges related to security, accuracy, and responsible AI governance. With careful implementation, ethical considerations, and international collaboration, DeepSeek and similar LLMs could enhance healthcare innovation, providing cost-effective, scalable AI solutions while ensuring human expertise remains at the forefront of patient care.

## Editorial

Introduction

During the previous couple of years, Generative Artificial Intelligence (GAI) has significantly advanced, particularly in healthcare applications. For example, OpenAI's ChatGPT evolved into the o1 model, with the o3 version announced for imminent release and excellent performance on math and science tests [1,2]. Similarly, Google's BERT transformed into Gemini, enhancing its capabilities, and other large language models (LLMs) continue to evolve and progress [3]. These proprietary models have demonstrated remarkable potential in clinical decision support, patient communication, and medical education, though the price tag could be elevated for some models [4,5]. Also, the use of LLMs in healthcare has many limitations. Studies have highlighted issues such as inherent biases and the phenomenon of "hallucinations," where models generate plausible but incorrect or nonsensical information [6,7]. These challenges raise concerns about reliability and ethical integrity in deploying such models in healthcare settings.

In January 2025, DeepSeek introduced DeepThink (R1), an innovative open-source LLM that has rapidly gained prominence throughout the world [8,9]. This editorial explores several potentials of this advanced LLM, highlighting several pros and cons for healthcare professionals and stakeholders (Table 1).

**Table 1:** Summary of DeepSeek DeepThink (R1)’s key advantages and challenges in healthcare. AI: Artificial Intelligence, GAI: generative artificial intelligence, GDPR: general data protection regulation, HIPAA: health insurance portability and accountability act, LLM: large language model.

Aspect	Pros	Cons
Open-source accessibility	Facilitates collaboration, allowing researchers and clinicians to refine models for specific needs.	May lead to unregulated modifications, creating risks in standardization and clinical reliability.
Cost efficiency	Lower computational costs compared to proprietary LLMs, making AI more accessible in resource-limited settings.	Still requires computational resources, and large-scale implementation may pose infrastructure challenges.
Customizability	Allows fine-tuning for region-specific applications, including localized healthcare challenges.	Without proper oversight, inconsistent modifications could reduce reliability in high-stakes medical scenarios.
Bias and data integrity	Open-source model enables continuous auditing and bias correction through community-driven improvements.	Risk of inheriting biases from training data; errors in model fine-tuning could perpetuate disparities in healthcare.
Hallucinations and accuracy	Adaptability to different tasks can help enhance accuracy when fine-tuned properly for specific medical use cases.	Like other LLMs, risks generating inaccurate or misleading medical information without proper human oversight.
Regulatory compliance	Potential to be adapted to local regulatory frameworks with proper governance.	Complex legal and ethical challenges in ensuring compliance with HIPAA, GDPR, and other data protection laws.
Security and privacy	Transparent development may allow for better oversight in ensuring the secure handling of patient data.	Open-source nature raises risks of unauthorized access and potential data leaks.
Clinical decision support	Enhances clinical decision-making by processing large datasets and identifying patterns that may be overlooked by human experts.	Final decision-making must remain human-led, as AI-driven recommendations cannot replace medical expertise.
Medical education	Provides innovative, AI-driven learning environments, including case simulations for medical trainees.	Requires significant efforts to validate AI-generated content for educational reliability.
Scalability	Scales well with emerging research needs, enabling rapid integration of the latest medical knowledge.	Widespread adoption may still face resistance from institutions that prefer proprietary models with structured support.
Continuous improvement	The model can theoretically continue refining its training using publicly available open-source data, enhancing its capabilities over time.	Potential risks in data reliability due to the lack of a centralized oversight mechanism for ongoing training.
Offline usage and privacy	Open-source accessibility allows DeepSeek to be used locally without requiring an internet connection, providing an additional layer of privacy and security for sensitive healthcare data.	Local deployment requires high computational power, which may not be feasible for all institutions.

Key differentiators in DeepSeek with DeepThink (R1)

Unlike other proprietary counterparts, DeepThink R1 offers free access to an advanced LLM model comparable to ChatGPT o1 [2,10]. A significant advantage of DeepThink R1 is its open-source nature, so users can visualize and customize the model’s source code [8]. Also, such open-source may facilitate continuous LLM learning through integration with publicly available datasets [11]. Such continuous learning capability may allow the LLM to stay updated with the latest scientific reasoning and medical knowledge; therefore, having more adaptability to emerging healthcare trends and potentially enhancing its performance over time.

DeepSeek offline usage in healthcare privacy and compliance

A prominent feature of DeepSeek's framework is its support for offline deployment [8]. This capability may enable healthcare institutions and stakeholders to adapt a local LLM and operate the model without an internet connection, thereby enhancing data privacy and security [12]. In an era where compliance with medical data regulations such as the Health Insurance Portability and Accountability Act (HIPAA) and the General Data Protection Regulation (GDPR) is paramount, the ability to process data locally without transmitting sensitive patient or healthcare facility information over the internet is a significant advantage [13]. This offline functionality may strengthen data security and enhance healthcare system confidentiality, in compliance with stringent regulatory data privacy requirements.

DeepSeek's data privacy

Despite its potential advantages, DeepSeek may face scrutiny regarding its data privacy practices. Notably, Italy's data protection authority has blocked access to DeepSeek, citing issues with the application's handling of personal data, including its collection, storage, and user notification processes [14]. Additionally, open data and security lapses may expose sensitive user data, chat histories, and system logs, as found by security researchers within “minutes,” without authentication requirements, further intensifying privacy concerns [15]. These incidents may lead to increased inquiries from international regulators and highlight the importance of robust data protection measures in GAI applications. Furthermore, even if a user deletes their account, it is unclear whether all personal data is permanently removed from DeepSeek's servers.

DeepSeek's compliance with Chinese regulations and global implications

Operating within China's regulatory framework, DeepSeek is subject to national laws that mandate data sharing with government authorities upon request [16]. This requirement may have global implications, particularly for international users concerned about the confidentiality of their data. The intersection of Chinese data regulations with global healthcare data privacy standards presents a complex challenge that warrants further research. Healthcare providers and patients worldwide must consider these factors when deciding to adopt online versus offline DeepSeek's technology, weighing the benefits of its advanced capabilities against potential risks to data privacy.

International scholars' innovations and discoveries through open-source advanced LLMs

The open-source nature of DeepSeek's DeepThink R1 may empower innovation among international scholars [9]. Researchers could leverage the model to develop customized applications tailored to specific medical fields, leading to inspiring discoveries and advancements at lower cost and faster pace, as the collaborative environment fostered by open-source platforms encourages the sharing of knowledge and resources [10]. This democratization of technology empowers institutions across varying resource levels to contribute to and benefit from cutting-edge AI developments in healthcare.

Medical education and DeepSeek: what's next?

In medical education, DeepSeek's advanced LLM may offer open-source applications with decreased financial costs [10]. Educators can utilize the model to create interactive learning tools, simulate complex clinical scenarios, and provide personalized tutoring to students. The model's ability to process and generate human-like text enables the development of realistic patient interactions, enhancing the training of future healthcare professionals. As open-source LLMs continue to evolve, they hold additional potential to reform medical education by providing scalable, accessible, and high-quality educational resources [17].

Open source GAI and patient information: exploring truth versus hallucinations

A vital area of ongoing research is the open-source LLMs’ handling of patient information, particularly distinguishing between accurate data and hallucinations. Ensuring the reliability of information generated by DeepSeek is essential for its safe application in clinical settings, and this is confirmed in the developer’s terms of use [18]. Researchers are encouraged to explore and compare various advanced LLMs, like DeepSeek’s DeepThink (R1) and OpenAI’s ChatGPT-o1, investigating various methods to minimize hallucinations and enhance the models’ accuracy, such as refining training datasets, implementing robust validation protocols, and developing algorithms to detect and correct erroneous outputs [19,20]. Addressing these challenges is fundamental to building trust among healthcare providers and patients in the use of AI-driven tools.

Ownership and use of AI-generated output: user, company, or both?

The question of who owns the content generated by DeepSeek remains a pertinent issue. According to DeepSeek's privacy policy, the company reserves the right to use data collected from users to improve its services, which may include utilizing user-generated content [18]. Unlike ChatGPT’s “Temporary Chat” option, which ensures that user interactions are neither stored nor used for model training, DeepSeek’s user-generated content may be retained and accessed by the company’s developers, with no option for healthcare professionals or institutions to opt out [18,21]. This raises concerns about patient data privacy and compliance with healthcare regulations such as HIPAA and GDPR.

Limitations and future opportunities

Despite the potential promising capabilities of DeepSeek’s DeepThink (R1), real-world data evaluating its performance in healthcare settings remains limited, with no established empirical benchmarks comparing its efficacy, accuracy, and reliability against proprietary models like ChatGPT and Gemini. Given the rapid advancements in AI research, some references in this editorial are sourced from preprints or non-peer-reviewed materials, as peer-reviewed studies on DeepSeek’s real-world applications are yet to come. Its adaptability to clinical decision-making and compliance with evolving regulatory frameworks also require further validation. However, the open-source nature of DeepSeek presents unique opportunities for interdisciplinary collaboration between healthcare professionals and computer scientists. Future research should focus on structured evaluations, large-scale clinical trials, and independent benchmarking to determine its effectiveness and safety in healthcare. Additionally, efforts should be directed toward validating its reliability, mitigating biases, and optimizing it for domain-specific tasks such as diagnostics, personalized medicine, and medical education. To ensure ethical and secure deployment, robust validation frameworks and AI governance models must be developed. By leveraging collaborative research and real-world testing, DeepSeek and similar models could be refined to better serve the evolving needs of the medical and scientific communities.

Conclusions

DeepSeek's DeepThink (R1) represents a significant advancement in the application of open-source and free GAI in healthcare. Its continuous learning capabilities, offline deployment options, and collaborative potential offer numerous potential benefits for healthcare providers and data scientists. However, addressing concerns related to data privacy, regulatory compliance, information accuracy, and content ownership is crucial and warrants the urgent attention of policymakers and international regulators. By navigating these challenges thoughtfully, DeepSeek and similar open-source LLMs could play a pivotal role in the future of healthcare, enhancing patient care, medical research, and education through responsible and innovative AI collaborations.
